# Workers’ Compensation Claims for Musculoskeletal Disorders Among Wholesale and Retail Trade Industry Workers — Ohio, 2005–2009

**Published:** 2013-06-07

**Authors:** Ibraheem Tarawneh, Mike Lampl, Dave Robins, Steve Wurzelbacher, Steve Bertke, Jennifer Bell, Alysha Meyers

**Affiliations:** Ohio Bur of Workers’ Compensation; Div of Surveillance, Hazard Evaluations, and Field Studies; Div of Safety Research, National Institute for Occupational Safety and Health; EIS Officer, CDC

Work-related musculoskeletal disorders (WMSDs) resulting from ergonomic hazards are common in the United States. Recent data from the Bureau of Labor Statistics (BLS) indicate that in 2011, one third of occupational injuries and illnesses resulting in lost time from work were WMSDs (1). Based on data from the 2010 BLS Survey of Occupational Injuries and Illnesses, a higher rate of WMSDs resulting in lost time from work occurred in the Wholesale and Retail Trade (WRT) industry compared with most other industries (2). To assess trends and identify WRT subsectors and subgroups associated with high rates of WMSD workers’ compensation claims, the Ohio Bureau of Workers’ Compensation (OBWC) and CDC analyzed OBWC claims data for single-location WRT employers in Ohio for the period 2005–2009. From 2005 to 2009, the rate of WMSD claims declined from 86.3 to 52.8 per 10,000 employees. The three WRT industry subsectors with the highest rates of WMSD claims were Merchant Wholesalers, Nondurable Goods; Furniture and Home Furnishings Stores; and Merchant Wholesalers, Durable Goods. Within those three WRT subsectors, the highest rates of WMSD claims were noted in five subgroups: furniture stores and wholesalers of alcoholic beverages, groceries and related products, metal and minerals, and motor vehicle parts. Providing recommendations for WMSD prevention is particularly important for these WRT subgroups.

OBWC is the largest of four state-run workers’ compensation systems in the United States where the state is the sole provider of workers’ compensation insurance.* Data for OBWC-insured, single-location† employers in the WRT industry were used for this report; subsectors and subsector groups were categorized according to the North American Industry Classification System (NAICS). With few exceptions, WMSD claims were defined according to BLS case definitions.§ Coded injury/illness diagnosis data and narrative text on causation were used to identify WMSD claims; a Bayesian auto-coding technique (3) used both data elements to identify WMSDs by using a “training” and “testing” set of manually coded claims. The sensitivity and specificity of this auto-coding technique when applied to a test set were 0.90 and 0.98, respectively. Auto-coded WMSD claims were flagged for manual, expert review when the injury/illness diagnosis was not a WMSD. Lost-time claims for WMSDs were defined as claims resulting in more than 7 days away from work. To calculate incidence rates, OBWC claims data were linked with denominator data (number of employees) from the Ohio Department of Jobs and Family Services by federal employer identification numbers. Trends in rates were tested using Poisson regression analysis. Disallowed and dismissed claims were excluded from all analyses.

In 2009, CDC identified 31,599 OBWC-insured, single-location employers in the WRT industry, employing at least 289,441 workers. Of those identified WRT employers, 13,930 (44%) were in the wholesale category of the industry. The proportion of all claims attributable to WMSDs was relatively stable at approximately 20% throughout 2005–2009; the proportion of WMSD lost-time claims decreased from 37.4% in 2005 to 31.8% in 2009 (p<0.05) (Table 1). During 2005–2009, the majority of claimants were men aged 25–54 years, who worked for employers with 11–249 employees. The greatest number of WMSD claims occurred in the WRT subsector Merchant Wholesalers, Durable Goods (Table 1).

The rate of WMSDs resulting in a claim or a lost-time claim decreased significantly from 2005 to 2009 for WRT industry employers overall but not for all WRT subsectors. Overall in the WRT industry, the respective rates of WMSD claims and lost-time WMSD claims per 10,000 employees decreased from 86.3 and 28.7 in 2005 to 52.8 and 14.1 in 2009 (Table 2). Employers with more employees tended to have higher rates of total and lost-time WMSD claims. During 2005–2009, lost-time WMSD claim rates per 10,000 employees for three WRT subsectors were among the highest five each year: Merchant Wholesalers, Nondurable Goods (29.2 in 2009); Furniture and Home Furnishings Stores (21.7); and Merchant Wholesalers, Durable Goods (15.5) (Figure, Table 2). The high lost-time WMSD rates in these three WRT subsectors were consistently attributable to high rates in five subgroups within the subsectors: wholesalers of alcoholic beverages (114.8 in 2009), grocery and related products (30.9), metal and minerals (28.0), and motor vehicle parts and supplies (25.4); and furniture stores (27.2).

## Editorial Note

Improved surveillance of work-related WMSDs is a national priority (4). This report demonstrates how workers’ compensation claims data can be used for public health surveillance. The results indicate that although the rate of WMSD claims (overall and lost-time) among workers employed by OBWC-insured employers declined from 2005 to 2009 for most WRT subsectors, workers in some subsectors experienced higher rates of WMSD claims than workers in other WRT subsectors. The factors responsible for the downward trends in WMSD claims in Ohio in the WRT industry are unclear. At the national level, a downward trend for incident WMSDs from 2005 to 2009 also has been observed (2). For all workers’ compensation claims and industry sectors, the National Council on Compensation Insurance has reported downward trends among many states since the 1990s (5), attributing the trends, at least in part, to 1) advances in automation, technology, and production; 2) an aging workforce (older workers tend to have fewer claims [6]); and 3) increased focus on workplace safety and loss control.

Workers in the WRT subsectors with the highest rates of workers’ compensation claims are exposed to physical risk factors for WMSDs such as overexertion or repetitive motion (7). Work tasks in subgroups among those with the highest claim rates within the WRT subsectors (e.g., furniture stores and wholesalers of alcoholic beverages) commonly include lifting and transporting large, heavy objects. The Occupational Safety & Health Administration has created ergonomic training tools that outline injury prevention activities for beverage delivery and grocery warehousing.¶ Certain interventions (e.g., stair-climbing dollies, keg-handling equipment, and forklifts) can reduce many but not all manual material-handling tasks in these subgroups.

The findings in this report are subject to at least three limitations. First, this report is only representative of smaller employers (<500 employees) with a single location in Ohio. Second, the Bayesian auto-coding method used to identify WMSD claims introduces the potential for misclassification. However, misclassification is not expected to create bias in WMSD rates by WRT subsector. Finally, studies have estimated that workers’ compensation claims data underreport work-related injuries and illnesses by 40%–80% (8–10). However, whereas underreporting of injuries and illnesses might reduce the size of claim rates, whether the differences observed among WRT subsectors or employers of different sizes were affected by underreporting is unknown.

The findings in this report suggest that the number and rate of WMSD claims declined from 2005 to 2009 among small WRT employers in Ohio, but relatively high rates of WMSD claims occurred among certain WRT subsectors and subgroups. Interventions to reduce exposure to ergonomic hazards in these subsectors and subgroups should continue to be developed and implemented to prevent WMSDs. Given the large workforce employed in the WRT industry, declines in the number of WMSDs could substantially reduce the number of workplace injuries and illnesses overall.

What is already known on this topic?

Workers in the Wholesale and Retail Trade (WRT) industry have more work-related musculoskeletal disorders (WMSDs) resulting in lost work days than do most other workers.

What is added by this report?

Based on an analysis of claims filed with the Ohio Bureau of Workers’ Compensation by single-location WRT employers, WMSD claims decreased from 86.3 per 10,000 employees in 2005 to 52.8 in 2009. The WRT industry subsectors with the highest rates of WMSD claims during 2005–2009 were Wholesalers, Nondurable Goods; Furniture and Home Furnishings Stores; and Wholesalers, Durable Goods. Within those three WRT subsectors, the highest rates of WMSD claims were noted in five subgroups: furniture stores and wholesalers of alcoholic beverages, groceries and related products, metal and minerals, and motor vehicle parts.

What are the implications for public health practice?

Although the rate of claims for WMSD resulting in lost work days has decreased in the WRT industry in Ohio, workers continue to experience WMSDs, and some WRT subsectors are experiencing higher rates of WMSD claims than others. Prevention efforts are most needed in the WRT subgroups, wholesalers of alcoholic beverages and groceries and related products.

## Figures and Tables

**FIGURE:**
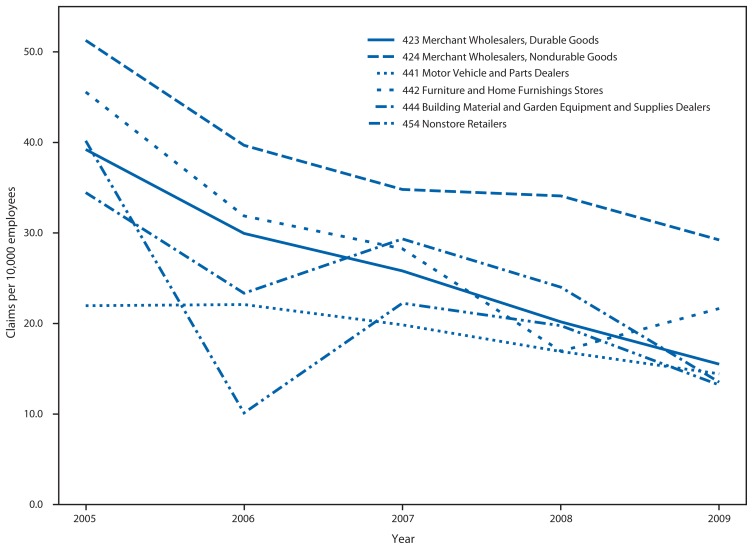
Rates of workers’ compensation claims for musculoskeletal disorders resulting in lost-time per 10,000 employees, among the WRT NAICS subsectors with the highest rates — Ohio, 2005–2009 **Abbreviations:** WRT = wholesale retail trade; NAICS = North American Industry Classification System.

**TABLE 1:** Number and percentage of WRT musculoskeletal disorder workers’ compensation claims, by claim status, age group, sex, single-location–employer size, and WRT NAICS code — Ohio, 2005, 2007, and 2009

Characteristic	2005				2007				2009			
		
	Total		LT		Total		LT		Total		LT	
					
	No.	(%)	No.	(%)	No.	(%)	No.	(%)	No.	(%)	No.	(%)
**WRT NAICS total**	**3,019**	**(21.6)**	**1,006**	**(37.4)**	**2,407**	**(21.1)**	**709**	**(33.8)**	**1,552**	**(20.3)**	**413**	**(31.8)**
**Age group (yrs)**												
14–17	7	(0.2)	0	0.0	12	(0.5)	2	(0.3)	4	(0.3)	0	0.0
18–19	78	(2.6)	19	(1.9)	40	(1.7)	6	(0.8)	19	(1.2)	2	(0.5)
20–24	313	(10.4)	66	(6.6)	250	(10.4)	41	(5.8)	137	(8.8)	21	(5.1)
25–34	787	(26.1)	215	(21.4)	600	(24.9)	150	(21.2)	368	(23.7)	71	(17.2)
35–44	880	(29.1)	297	(29.5)	706	(29.3)	223	(31.5)	420	(27.1)	113	(27.4)
45–54	685	(22.7)	283	(28.1)	544	(22.6)	190	(26.8)	391	(25.2)	131	(31.7)
55–64	244	(8.1)	116	(11.5)	229	(9.5)	89	(12.6)	199	(12.8)	69	(16.7)
≥65	24	(0.8)	9	(0.9)	26	(1.1)	8	(1.1)	14	(0.9)	6	(1.5)
Unknown	1	(0.0)	1	(0.1)	0	(0.0)	0	(0.0)	0	(0.0)	0	(0.0)
**Sex**												
Female	604	(20.0)	196	(19.5)	461	(19.2)	119	(16.8)	309	(19.9)	69	(16.7)
Male	2392	(79.2)	809	(80.4)	1919	(79.7)	589	(83.1)	1233	(79.4)	343	(83.1)
Unknown	23	(0.8)	1	(0.1)	27	(1.1)	1	(0.1)	10	(0.6)	1	(0.2)
**Employer size (no. of employees)**												
1–10	418	(13.8)	164	(16.3)	325	(13.5)	113	(15.9)	242	(15.6)	93	(22.5)
11–49	1104	(36.6)	373	(37.1)	838	(34.8)	258	(36.4)	508	(32.7)	132	(32.0)
50–249	1251	(41.4)	378	(37.6)	1044	(43.4)	277	(39.1)	642	(41.4)	142	(34.4)
≥250	237	(7.9)	86	(8.5)	192	(8.0)	58	(8.2)	137	(8.8)	41	(9.9)
Unknown	9	(0.3)	5	(0.5)	8	(0.3)	3	(0.4)	23	(1.5)	5	(1.2)
**WRT NAICS code**												
423 Merchant Wholesalers, Durable Goods	1008	(33.4)	333	(33.1)	787	(32.7)	221	(31.2)	463	(29.8)	115	(27.8)
424 Merchant Wholesalers, Nondurable Goods	559	(18.5)	207	(20.6)	468	(19.4)	137	(19.3)	376	(24.2)	101	(24.5)
425 Wholesale Electronic Markets and Agents and Brokers	119	(3.9)	31	(3.1)	101	(4.2)	19	(2.7)	76	(4.9)	20	(4.8)
441 Motor Vehicle and Parts Dealers	367	(12.2)	122	(12.1)	327	(13.6)	106	(15.0)	222	(14.3)	68	(16.5)
442 Furniture and Home Furnishings Stores	144	(4.8)	50	(5.0)	101	(4.2)	27	(3.8)	50	(3.2)	16	(3.9)
443 Electronics and Appliance Stores	46	(1.5)	10	(1.0)	29	(1.2)	7	(1.0)	26	(1.7)	4	(1.0)
444 Building Material and Garden Equipment and Supplies Dealers	220	(7.3)	69	(6.9)	155	(6.4)	54	(7.6)	81	(5.2)	20	(4.8)
445 Food and Beverage Stores	226	(7.5)	79	(7.9)	182	(7.6)	63	(8.9)	104	(6.7)	30	(7.3)
446 Health and Personal Care Stores	18	(0.6)	5	(0.5)	14	(0.6)	4	(0.6)	18	(1.2)	3	(0.7)
447 Gasoline Stations	52	(1.7)	17	(1.7)	44	(1.8)	18	(2.5)	30	(1.9)	12	(2.9)
448 Clothing and Clothing Accessories Stores	48	(1.6)	14	(1.4)	38	(1.6)	9	(1.3)	19	(1.2)	4	(1.0)
451 Sporting Goods, Hobby, Book, and Music Stores	31	(1.0)	8	(0.8)	20	(0.8)	4	(0.6)	14	(0.9)	3	(0.7)
452 General Merchandise Stores	6	(0.2)	3	(0.3)	14	(0.6)	3	(0.4)	9	(0.6)	2	(0.5)
453 Miscellaneous Store Retailers	80	(2.6)	22	(2.2)	60	(2.5)	18	(2.5)	26	(1.7)	6	(1.5)
454 Nonstore Retailers	95	(3.1)	36	(3.6)	67	(2.8)	19	(2.7)	38	(2.4)	9	(2.2)

**Abbreviations:** WRT = wholesale retail trade; NAICS = North American Industry Classification System; LT = lost-time claims (excluding claims for which the size of the employer was unknown).

**TABLE 2:** Rate and trend of musculoskeletal disorder workers’ compensation claims per 10,000 employees, by claim status, WRT NAICS code, and single-location–employer size — Ohio, 2005–2009

WRT NAICS code employer size (no. of employees)	2005		2009		Trend analysis*					

					Total			LT		
			
	Total	LT	Total	LT	Slope	(95% CLs)	p-value	Slope	(95% CLs)	p-value
**WRT NAICS total**	**86.3**	**28.7**	**52.8**	**14.1**	**−0.12**	**(−0.14, −0.10)**	**<0.001**	**−0.17**	**(−0.20, −0.13)**	**<0.001**
**Employer size**										
1–10	43.8	17.2	27.6	10.6	−0.12	(−0.13, −0.11)	<0.001	−0.15	(−0.21, −0.08)	<0.001
11–49	80.3	27.1	44.1	11.5	−0.15	(−0.16, −0.13)	<0.001	−0.19	(−0.24, −0.15)	<0.001
50–249	127.1	38.4	86.5	19.1	−0.09	(−0.12, −0.06)	<0.001	−0.14	(−0.22, −0.07)	<0.001
≥250	135.2	49.1	109.8	32.9	−0.05	(−0.08, −0.02)	0.003	−0.11	(−0.22, 0.00)	0.048
**423 Merchant Wholesalers, Durable Goods**	119.3	39.2	62.7	15.5	−0.15	(−0.17, −0.13)	<0.001	−0.22	(−0.25, −0.20)	<0.001
1–10	61.6	25.0	31.0	10.2	−0.17	(−0.18, −0.16)	<0.001	−0.27	(−0.40, −0.15)	<0.001
11–49	110.4	39.3	56.2	12.8	−0.16	(−0.19, −0.13)	<0.001	−0.25	(−0.32, −0.17)	<0.001
50–249	165.9	48.4	98.8	25.1	−0.12	(−0.14, −0.10)	<0.001	−0.16	(−0.26, −0.06)	0.002
≥250	208.7	51.1	107.1	17.1	−0.12	(−0.24, 0.01)	0.066	−0.36	(−0.58, −0.14)	0.001
**424 Merchant Wholesalers, Nondurable Goods**	138.9	51.3	108.8	29.2	−0.07	(−0.08, −0.05)	<0.001	−0.13	(−0.18, −0.09)	<0.001
1–10	53.7	21.2	45.6	21.3	−0.06	(−0.15, 0.02)	0.150	−0.06	(−0.22, 0.10)	0.478
11–49	112.5	40.6	70.3	12.2	−0.15	(−0.21, −0.08)	<0.001	−0.31	(−0.41, −0.20)	<0.001
50–249	206.1	66.9	154.6	34.5	−0.06	(−0.10, −0.03)	<0.001	−0.13	(−0.21, −0.05)	0.001
≥250	156.6	77.5	234.2	100.4	0.09	(0.01, 0.18)	0.032	0.07	(−0.02, 0.17)	0.136
**425 Wholesale Electronic Markets and Agents and Brokers**	61.4	16.3	37.4	10.5	−0.13	(−0.22, −0.05)	0.001	−0.16	(−0.29, −0.03)	0.017
1–10	35.2	7.9	23.6	9.0	−0.11	(−0.26, 0.04)	0.154	0.01	(−0.18, 0.20)	0.916
11–49	72.9	24.3	47.0	15.0	−0.12	(−0.24, 0.00)	0.060	−0.22	(−0.55, 0.12)	0.211
50–249	124.2	24.1	61.1	10.2	−0.21	(−0.44, 0.02)	0.079	−0.26	(−0.58, 0.05)	0.105
≥250	55.2	20.1	11.6	0.0	−0.25	(−0.61, 0.12)	0.184	−0.55	(−1.44, 0.35)	0.231
**441 Motor Vehicle and Parts Dealers**	66.1	22.0	46.7	14.4	−0.09	(−0.12, −0.06)	<0.001	−0.10	(−0.15, −0.06)	<0.001
1–10	69.5	27.8	42.3	18.3	−0.10	(−0.21, 0.01)	0.062	−0.14	(−0.22, −0.05)	0.002
11–49	58.2	22.1	35.1	15.9	−0.10	(−0.19, 0.00)	0.048	−0.08	(−0.14, −0.02)	0.008
50–249	67.8	20.9	55.3	12.0	−0.08	(−0.16, 0.00)	0.040	−0.13	(−0.16, −0.10)	<0.001
≥250	128.6	12.2	110.7	6.9	−0.05	(−0.13, 0.03)	0.210	−0.12	(−0.52, 0.29)	0.573
**442 Furniture and Home Furnishings Stores**	133.0	45.6	67.7	21.7	−0.17	(−0.25, −0.09)	<0.001	−0.23	(−0.34, −0.12)	<0.001
1–10	55.5	26.7	36.1	22.2	−0.16	(−0.36, 0.04)	0.126	−0.14	(−0.47, 0.19)	0.405
11–49	152.2	42.9	35.1	15.6	−0.33	(−0.42, −0.24)	<0.001	−0.29	(−0.51, −0.08)	0.007
50–249	296.2	107.1	192.3	21.4	−0.07	(−0.26, 0.12)	0.480	−0.28	(−0.72, 0.16)	0.208
≥250	—	—	341.3	68.3	NC	NC	NC	NC	NC	NC
**443 Electronics and Appliance Stores**	58.9	12.8	38.6	5.9	−0.13	(−0.25, −0.02)	0.024	−0.19	(−0.44, 0.06)	0.141
1–10	33.4	13.4	39.1	0.0	0.01	(−0.19, 0.22)	0.896	−0.58	(−1.17, 0.01)	0.053
11–49	75.3	19.6	39.4	9.8	−0.20	(−0.38, −0.01)	0.034	−0.13	(−0.46, 0.20)	0.452
50–249	92.7	0.0	54.1	12.0	−0.14	(−0.37, 0.08)	0.206	0.41	(−0.29, 1.12)	0.247
≥250	0.0	0.0	0.0	0.0	0.34	(−1.44, 2.12)	0.709	NC	NC	NC
**444 Building Material and Garden Equipment and Supplies Dealers**	109.8	34.5	54.1	13.5	−0.17	(−0.22, −0.11)	<0.001	−0.16	(−0.26, −0.07)	0.001
1–10	58.2	21.6	33.1	11.0	−0.16	(−0.28, −0.04)	0.011	−0.18	(−0.39, 0.02)	0.082
11–49	119.3	37.8	57.9	12.1	−0.17	(−0.25, −0.10)	<0.001	−0.17	(−0.30, −0.03)	0.014
50–249	167.5	45.9	99.0	26.1	−0.10	(−0.2, 0.00)	0.052	−0.08	(−0.27, 0.11)	0.384
≥250	—	—	—	—	NC	NC	NC	NC	NC	NC
**445 Food and Beverage Stores**	63.7	22.4	34.5	10.0	−0.14	(−0.19, −0.09)	<0.001	−0.16	(−0.24, −0.07)	<0.001
1–10	36.8	10.9	26.2	11.5	−0.05	(−0.17, 0.06)	0.385	0.02	(−0.16, 0.21)	0.829
11–49	50.2	19.6	27.5	6.9	−0.13	(−0.22, −0.05)	0.002	−0.24	(−0.40, −0.08)	0.003
50–249	97.1	36.2	54.6	10.2	−0.12	(−0.19, −0.04)	0.002	−0.20	(−0.34, −0.06)	0.006
≥250	172.4	30.4	39.1	29.3	−0.38	(−0.55, −0.21)	<0.001	−0.09	(−0.38, 0.21)	0.571
**446 Health and Personal Care Stores**	17.0	4.7	17.4	2.9	−0.02	(−0.16, 0.13)	0.839	−0.11	(−0.44, 0.22)	0.512
1–10	6.0	3.0	6.3	3.2	−0.21	(−0.75, 0.33)	0.452	−0.16	(−0.97, 0.66)	0.705
11–49	13.5	1.9	15.6	4.4	0.03	(−0.24, 0.30)	0.813	0.47	(−0.23, 1.17)	0.188
50–249	54.3	18.1	56.5	0.0	−0.04	(−0.25, 0.17)	0.723	−0.53	(−1.16, 0.11)	0.105
≥250	0.0	0.0	7.8	0.0	−0.01	(−0.44, 0.42)	0.968	−0.31	(−1.11, 0.48)	0.440
**447 Gasoline Stations**	41.4	13.5	30.6	12.3	−0.07	(−0.16, 0.03)	0.185	−0.02	(−0.19, 0.15)	0.791
1–10	36.3	21.0	28.3	12.1	−0.02	(−0.17, 0.14)	0.823	−0.08	(−0.32, 0.17)	0.528
11–49	21.7	4.0	24.5	7.4	0.00	(−0.16, 0.16)	0.966	−0.01	(−0.29, 0.28)	0.964
50–249	86.2	17.2	99.0	59.4	−0.03	(−0.25, 0.19)	0.797	0.26	(−0.21, 0.73)	0.278
≥250	135.1	19.3	37.9	0.0	NC	NC	NC	NC	NC	NC
**448 Clothing and Clothing Accessories Stores**	38.2	11.1	21.5	4.5	−0.18	(−0.29, −0.08)	0.001	−0.15	(−0.36, 0.05)	0.145
1–10	6.1	4.1	2.3	2.3	−0.16	(−0.51, 0.19)	0.378	0.03	(−0.53, 0.59)	0.918
11–49	19.2	5.5	11.6	3.9	−0.25	(−0.52, 0.02)	0.071	−0.04	(−0.54, 0.45)	0.864
50–249	121.6	32.0	75.4	10.1	−0.16	(−0.29, −0.03)	0.014	−0.22	(−0.48, 0.04)	0.101
≥250	0.0	0.0	—	—	NC	NC	NC	NC	NC	NC
**451 Sporting Goods, Hobby, Book, and Music Stores**	32.6	8.4	17.7	3.8	−0.17	(−0.30, −0.03)	0.016	−0.15	(−0.41, 0.10)	0.241
1–10	17.2	4.9	15.2	3.0	−0.18	(−0.43, 0.07)	0.154	−0.35	(−0.9, 0.19)	0.204
11–49	26.0	7.8	13.7	6.9	−0.19	(−0.43, 0.05)	0.121	−0.07	(−0.43, 0.30)	0.717
50–249	105.9	22.7	11.3	0.0	−0.30	(−0.55, −0.04)	0.022	−0.06	(−0.56, 0.44)	0.817
≥250	0.0	0.0	48.4	0.0	0.88	(−0.05, 1.81)	0.065	NC	NC	NC
**452 General Merchandise Stores**	28.1	14.0	64.8	14.4	0.09	(−0.13, 0.31)	0.427	−0.10	(−0.55, 0.36)	0.680
1–10	38.8	25.9	48.7	0.0	0.05	(−0.40, 0.51)	0.818	−0.79	(−1.95, 0.37)	0.184
11–49	29.9	10.0	43.9	14.6	0.00	(−0.34, 0.34)	0.995	−0.01	(−0.64, 0.61)	0.973
50–249	0.0	0.0	333.3	111.1	0.45	(0.03, 0.86)	0.034	0.74	(−0.42, 1.89)	0.211
≥250	—	—	—	—	NC	NC	NC	NC	NC	NC
**453 Miscellaneous Store Retailers**	40.5	11.1	18.6	4.3	−0.16	(−0.24, −0.07)	<0.001	−0.17	(−0.32, −0.01)	0.035
1–10	25.6	8.5	12.1	6.7	−0.19	(−0.35, −0.03)	0.019	−0.03	(−0.29, 0.23)	0.846
11–49	48.5	10.0	22.0	2.0	−0.16	(−0.29, −0.03)	0.016	−0.16	(−0.40, 0.09)	0.203
50–249	65.3	20.8	38.7	0.0	−0.07	(−0.23, 0.10)	0.425	−0.31	(−0.65, 0.03)	0.074
≥250	—	—	—	—	NC	NC	NC	NC	NC	NC
**454 Nonstore Retailers**	106.0	40.2	55.8	13.2	−0.14	(−0.22, −0.05)	0.001	−0.22	(−0.37, −0.06)	0.006
1–10	83.2	41.6	22.6	0.0	−0.21	(−0.43, 0.00)	0.054	−0.50	(−0.90, −0.09)	0.017
11–49	80.9	17.6	56.3	18.8	−0.09	(−0.26, 0.07)	0.268	0.12	(−0.19, 0.43)	0.455
50–249	91.1	30.4	68.4	16.1	−0.03	(−0.17, 0.12)	0.720	−0.04	(−0.31, 0.24)	0.784
≥250	185.2	84.7	117.9	23.6	−0.17	(−0.34, −0.01)	0.037	−0.59	(−0.98, −0.21)	0.002

**Abbreviations:** WRT = wholesale retail trade; NAICS = North American Industry Classification System; LT = lost-time claims (excluding claims for which the size of the employer was unknown); CLs = confidence limits; NC = not calculable.

* Trend analysis is based on 5 years of data. Trends were not calculable where rates were missing for ≥1 years.

## Footnotes

* All public Ohio employers and private employers (except sole proprietorships or partnerships) with fewer than 500 employees must participate in the OBWC system. Other private employers have the option to self-insure for workers’ compensation insurance. OBWC provides workers’ compensation insurance for approximately two thirds of Ohio workers but a smaller proportion of WRT workers.

† OBWC claims cannot be linked to a particular employer if the policy includes more than one location. Therefore, these analyses are confined to single-location employers. In 2009, among identified OBWC-insured WRT employers, employees at 31,599 single-location employers filed 7,661 workers’ compensation claims and employees at 882 multiple-location employers filed 3,441 claims.

§ BLS case definitions are available at http://www.bls.gov/iif/oshdef.htm. This analysis used a definition for musculoskeletal disorders that included Raynaud’s phenomenon, tarsal tunnel syndrome, and herniated spinal discs, similar to the revised BLS case definition (2011 and forward). Also, the work-related musculoskeletal disorders case definition used in this analysis excluded events or exposures resulting from a single episode of overexertion/bodily reaction from climbing down, stepping down, or walking or running without other incident (i.e., missteps).

¶ Available at http://www.osha.gov/dts/osta/oshasoft/index.html.
